# Iodine nanoparticle radiotherapy of human breast cancer growing in the brains of athymic mice

**DOI:** 10.1038/s41598-020-72268-0

**Published:** 2020-09-24

**Authors:** James F. Hainfeld, Sharif M. Ridwan, F. Yaroslav Stanishevskiy, Henry M. Smilowitz

**Affiliations:** 1 Nanoprobes, Inc., 95 Horseblock Rd., Unit 1, Yaphank, NY 11980 USA; 2 Department of Cell Biology, University of Connecticut Health Center, 263 Farmington Ave., Farmington, CT 06030 USA

**Keywords:** Nanoparticles, Breast cancer, Cancer imaging, Cancer therapy, Metastasis, Cancer, Breast cancer, Cancer imaging, CNS cancer, Metastasis, Nanobiotechnology, Nanomedicine, Breast cancer, Cancer imaging, Cancer therapy, CNS cancer, Metastasis, Biotechnology, Cancer, Oncology, Nanoscience and technology

## Abstract

About 30% of breast cancers metastasize to the brain; those widely disseminated are fatal typically in 3–4 months, even with the best available treatments, including surgery, drugs, and radiotherapy. To address this dire situation, we have developed iodine nanoparticles (INPs) that target brain tumors after intravenous (IV) injection. The iodine then absorbs X-rays during radiotherapy (RT), creating free radicals and local tumor damage, effectively boosting the local RT dose at the tumor. Efficacy was tested using the very aggressive human triple negative breast cancer (TNBC, MDA-MB-231 cells) growing in the brains of athymic nude mice. With a well-tolerated non-toxic IV dose of the INPs (7 g iodine/kg body weight), tumors showed a heavily iodinated rim surrounding the tumor having an average uptake of 2.9% iodine by weight, with uptake peaks at 4.5%. This is calculated to provide a dose enhancement factor of approximately 5.5 (peaks at 8.0), the highest ever reported for any radiation-enhancing agents. With RT alone (15 Gy, single dose), all animals died by 72 days; INP pretreatment resulted in longer-term remissions with 40% of mice surviving 150 days and 30% surviving > 280 days.

## Introduction

As of 2018, cancer has become the leading cause of death in the U.S. and developed countries^1^. Cancers are relentless when in the brain. Once there, they account for about 20% of all cancer deaths. Brain tumors are also the leading cause of cancer deaths in children^2^. In adults, primary brain tumors (tumors that originate in the brain) include the most aggressive glioblastoma multiform (GBM) which after optimal therapy nearly always recurs after which most patients do not survive beyond one year^3,4^. More prevalent are brain tumors from other parts of the body (primarily from breast and lung, but also from colon, kidney and melanoma) that metastasize to the brain and account for about 90% of all brain tumors. About 30% of people with cancer develop brain metastases. Untreated brain metastases are associated with a median survival time of only about one month due to increasing intracranial pressure and cerebral herniation^5,6^. Because most patients present with multiple metastases, whole-brain radiotherapy (WBRT) with corticosteroids is the most common form of treatment, but this only increases the median survival 3–6 months^5,6^. Surgical resection and stereotactic radiosurgery are performed in selected patients^5^. However, surgery is generally not indicated since there are typically multiple lesions (some microscopic) scattered throughout the brain. Drugs have not been effective due to the tight blood brain barrier and blood tumor barrier hindering delivery, and also are ejected from tumor cells by biochemical efflux pumps. While WBRT can temporarily slow progression, sufficient dose cannot be given to eradicate the cancerous lesions without excessive normal brain damage. In summary, the effective treatment of brain metastases is a significant unmet medical need.

**Figure 1:**
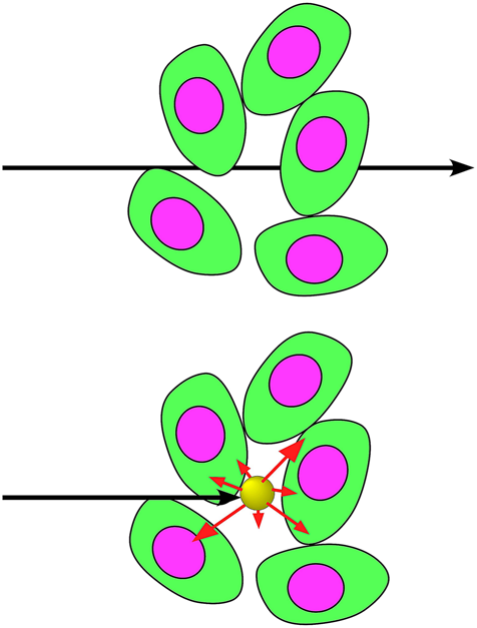
X-rays that would normally pass through tissue are instead absorbed by a high atomic number material and the energy deposited locally. Figure from reference^7^.

The approach we propose here is to load tumors with a high atomic number (Z) element that absorbs X-rays. Upon irradiation, X-rays that would have passed through are captured and the energy is deposited locally, in effect boosting the local dose (Fig. 1).

Physical dose enhancement using high atomic number elements has been known for many years^8^. In 1980 Matsudaira et al. measured a radiation dose-enhancement effect of iodine on cultured cells^9^. Iododeoxyuridine was used to incorporate iodine into cells resulting in about a threefold radiation enhancement effect^10^. Use of iodine contrast media was pioneered by Norman, Adams, Rose, Mello and collaborators as early as 1977^11^. They found direct injection into experimental subcutaneous Ehrlich ascites tumors in mice followed by radiotherapy (RT) using 100kVp X-rays suppressed the growth of 80% of the tumors^12^. After intravenous injection of iodine contrast medium into 47 dogs with spontaneous brain tumors, followed by RT (140kVp from a CT scanner), they found 53% longer survival^13^. More recently, rats^14^ and human patients^15^ with gliomas were treated with stereotactic synchrotron RT shortly after IV injection of iodine contrast medium. A drawback with this approach has been the short blood half-life of the iodine contrast medium: 45 s, followed by a slower phase half-life of ~ 13 min^16^. The clearance is so rapid there is not enough time for the iodine to build up significantly in the tumor or differentially clear normal surrounding tissues. Nanoparticles can have a longer blood half-life and better specific tumor uptake. Intravenously injected gold nanoparticles were shown to enhance radiation dose to tumors and result in significant life extension in subcutaneous tumors^17–19^ and in brain tumors in mice^20^. Other high atomic number nanoparticles are also being explored including gadolinium^21^, hafnium^22^, and bismuth^23^. Although gold nanoparticles appear to work well, they showed some disadvantages for human use: very poor whole body clearance^24^, long-term skin discoloration^25^, and high cost. We therefore constructed iodine nanoparticles that are very well tolerated with no demonstrable toxicity, have a pathway for biodegradation, a long blood half-life (40 h), an appropriate size for tumor uptake (20 nm), and are lower in cost^25^. These were used previously to treat athymic nude mice with an advanced intracerebral human glioma resulting in two to threefold median life extensions^7^. In the study presented here, using a human triple-negative breast tumor growing in the athymic mouse brain, median survival with INP-RT also increased by about two-fold, but significantly, 40% of the mice experienced from 7 to > 20-fold longer life extensions compared to RT only.

## Results

**Figure 2:**
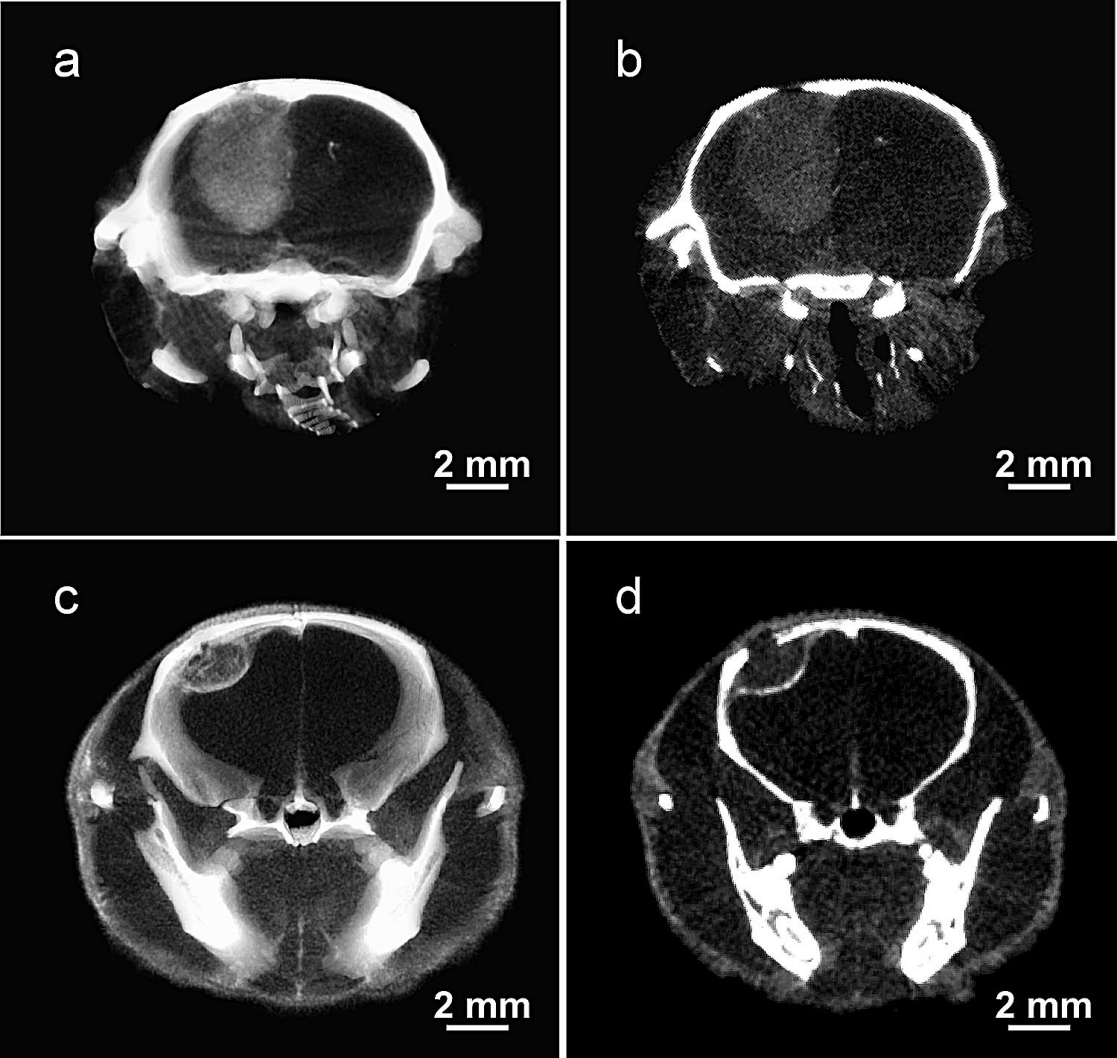
MicroCT images of brain tumors 3 days after iv injection of INPs. (**a**) and (**b**) are the U87 glioma and (**c**) and (**d**) are the 231-TNBC tumor. (**a**) and (**c**) include the whole tumor in a thick section (1.8 mm) and (**b**) and (**d**) are 65 μm thin sections through the tumor centers. Although the iodine doses were different (3.5 g I/kg for glioma, 7 g I/kg for TNBC), the images illustrate the considerable distribution differences). Data displayed using Amira 3.1 software (https://www.thermofisher.com/us/en/home/industrial/electron-microscopy/electron-microscopy-instruments-workflow-solutions/3d-visualization-analysis-software/amira-advanced-image-processing-quantification.html).

Brain tumors in athymic mice were initiated by intracerebral injection of human tumor cells, either glioma (U87), or triple negative breast cancer (TNBC, MDA-MB-231). Once tumors have been established, the iodine nanoparticles (INPs) were intravenously injected. MicroCT revealed specific tumor uptake with both tumor lines (Fig. 2).

However, there was a striking difference in the growth and uptake patterns. Gliomas had a uniformly heterogeneous uptake pattern (Fig. 2a) whereas the breast tumor had a higher iodine uptake at its periphery (Fig. 2c). Thin slices through the center of these tumors revealed a similar pattern. The glioma had a uniformly heterogeneous and diffuse uptake showing no clear preference for the tumor edge (Fig. 2b), whereas the breast tumor again showed a highly concentrated iodine uptake at the tumor periphery (Fig. 2d). Quantification (by calibrated microCT) of the uptake in the breast tumor after a non-toxic injection of 7 g Iodine/kg is given in Table 1.

**Table 1:** Iodine uptake and calculated Dose Enhancement Factor (DEF) at 24 and 72 h after iv injection of 7 g I/kg INPs for TNBC growing in brains of mice. Regions measured: inside tumor rim, tumor rim, peak regions of rim, expressed as mean ± standard deviation.

	24 h avg inside	24 h avg avg rim	24 h peak rim	72 h avg inside	72 h avg rim	72 h peak rim
%iodine	0.99 ± 0.13	1.82 ± 0.22	3.09 ± 0.11	1.13 ± 0.35	2.9 ± 0.54	4.52 ± 0.07
DEF	2.58	3.82	5.75	2.77	5.48	8.01

Significantly, the tumor-to-non-tumor ratio measured at 72 h was found to be 19.8:1. The Dose Enhancement Factor (DEF) was estimated from the percent iodine in the tumor based on previous Monte Carlo treatments^26,27^. Although approximate, values hitting as high as 4.5% iodine in the peripheral rim of the tumor indicate that the radiation dose delivered there may be multiplied by a factor of 8. These levels are higher than we previously reported for the U87 human glioma model^25^. An expectation is that this high DEF may then produce significant benefit in a radiotherapy treatment.

**Figure 3:**
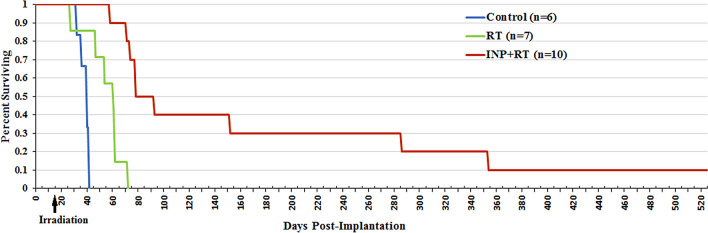
Survival graph showing no treatment (blue), RT only (15 Gy, green), and INP + RT (red).

Three groups of athymic nude mice bearing advanced intracerebral 231 tumors were subsequently studied: no treatment, RT only (15 Gy, single dose), and INP (7 g I/kg, IV) followed 24 h later with the same RT (15 Gy). Resulting survival is shown in Fig. 3.

RT alone provided a 20 day median life extension. RT + INP provided a 45-day median life extension (2.25-fold compared to RT). Remarkably, 40% of the mice experienced extraordinary life extensions. One of the four mice required euthanasia at 152 days (> sevenfold life extension compared to RT only). Three mice lived to 286 days (9.5 months, > 13-fold life extensions), 2 survived 1 year (17-fold life extension) and 1 mouse is still alive at 520 days (1.4 years, > 25-fold life extension). The normal lifespan of a mouse is 2 years.

**Figure 4:**
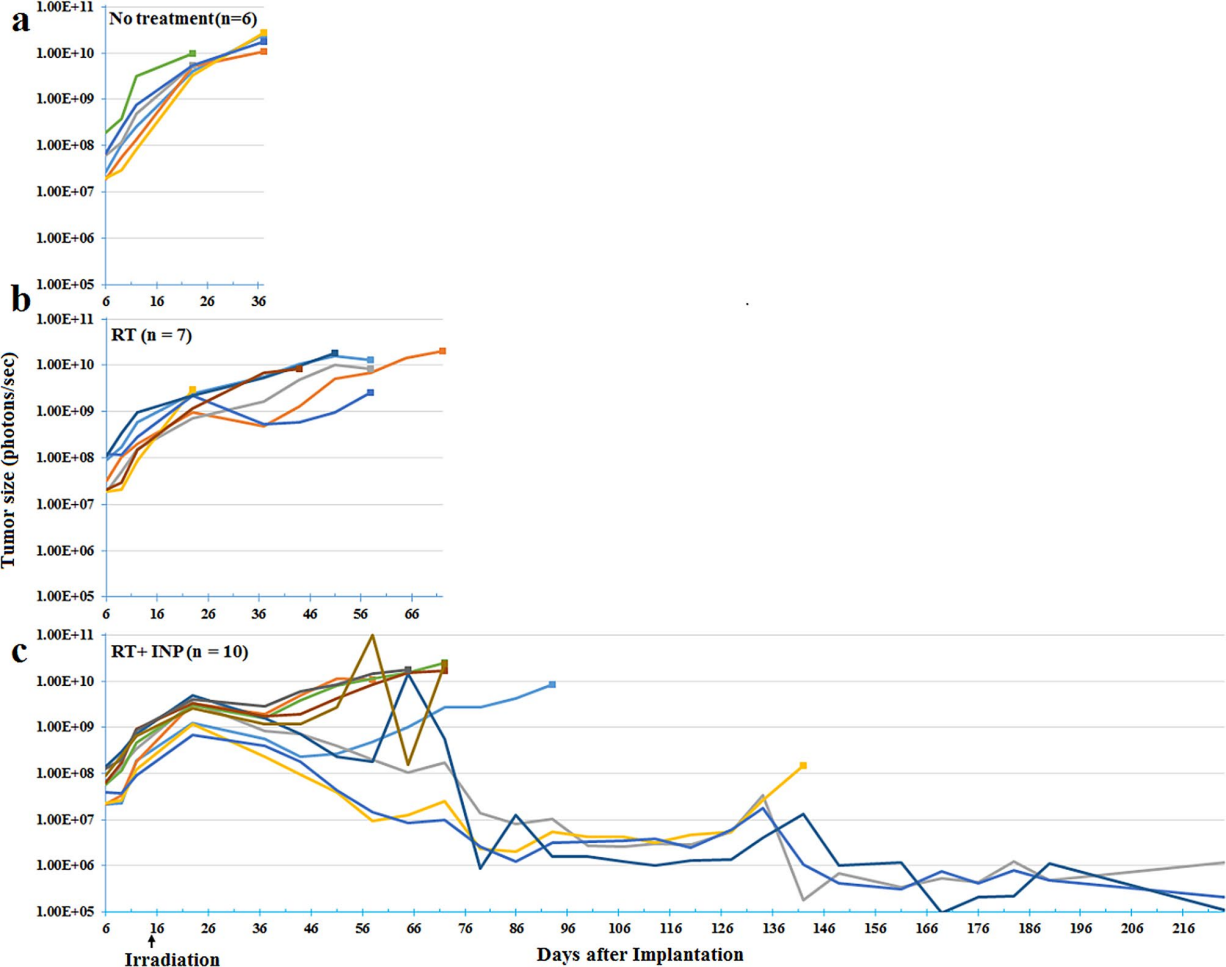
IVIS luciferase imaging reporting on the number of viable tumor cells. Each line represents one mouse. (**a**): no treatment; (**b**): RT only; (**c**): INP + RT.

The 231 TNBC cells had been transduced with luciferase so that tumor size could be followed using IVIS (In Vivo Imaging System). The light emitted is proportional to the number of viable tumor cells. These measurements are shown in Fig. 4.

With no treatment (a), tumors continued to grow unabated until the mice became sick and required euthanasia, with a median survival of 39 days. With RT (b), there was a slower growth rate for about 8 days followed by a somewhat even slower rate, but still ending in 100% tumor overgrowth, with a median survival of 61 days. For the INP + RT group (c), the slower 8-day period of growth after irradiation was similar to the RT group. However, after that, all tumors shrank for about 15 days. Subsequently, 60% began growing at a slow rate ending in tumor overgrowth, with median survival extended to 85 days. Three of the 10 mice had IVIS signals that decreased to the background count rate for mice injected with luciferin but having no tumor. However, the small number of residual tumor cells eventually led to tumor recurrence in all but one mouse, still alive at 520 days.

## Discussion

Cancers that metastasize to the brain have a dismal prognosis. By that time, metastases to other parts of the body are also poorly controlled. Nevertheless, improvement in treating the brain metastases may be of benefit to some patients.

**Figure 5:**
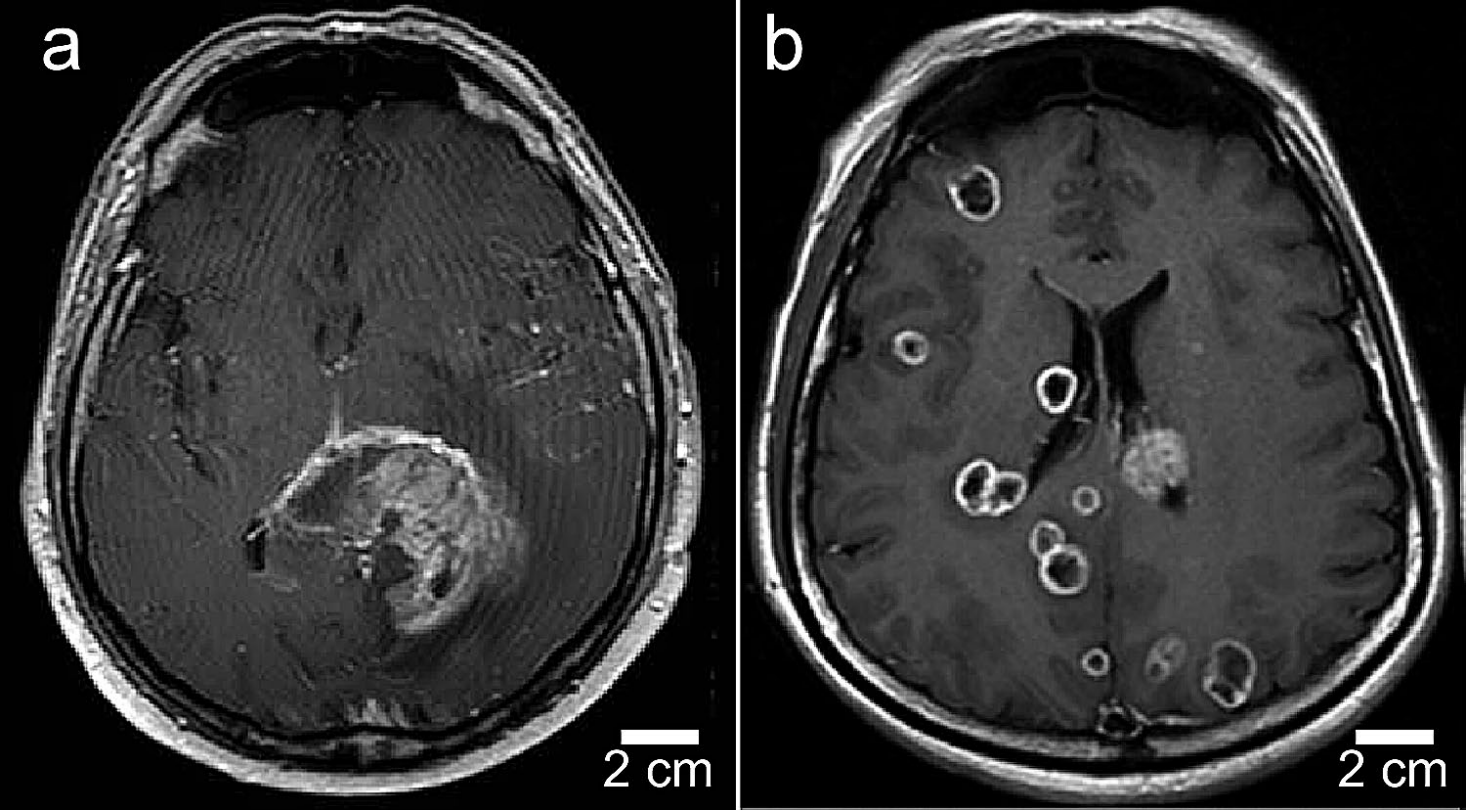
Human T1-MRI of glioblastoma multiform (**a**) and TNBC breast metastasis (**b**). Mets have rings of enhancement showing more active growth. GBM is more heterogeneous and diffuse with extensive edema and infiltrative tumor. Figures from references^28,29^, courtesy of John Wiley and Sons, Copyright 2017 by the American Society of Neuroimaging, and Taylor & Francis Ltd., www.tandofonline.com).

A novel finding of this study is that IV injected INPs heavily load the perimeter of an advanced breast cancer growing in the brains of mice in stark contrast to the more diffuse and lower accumulations seen in a glioma model (Fig. 2). This indicates a different pattern of tumor growth which is also seen in humans (Fig. 5).

For our study we used the same types of human tumor shown in Fig. 5 implanted into mouse brains (human glioma and human TNBC) and observed similar growth patterns. Gliomas spread by migrating along blood vessels and white matter tracts associated with edema^30–32^. Intracerebral TNBCs seem to grow in a more confined way with a more active growing edge. We also saw this pattern in mice with subcutaneous breast tumors after gold nanoparticle injections^18^. If this growing edge can be more highly damaged by RT, it could presumably further reduce the supply of oxygen and nutrients to the tumor cells within, resulting in their demise. We hypothesize this mechanism accounts for the near eradication of the human TNBC tumors in some of the mice seen in this study.

**Figure 6:**
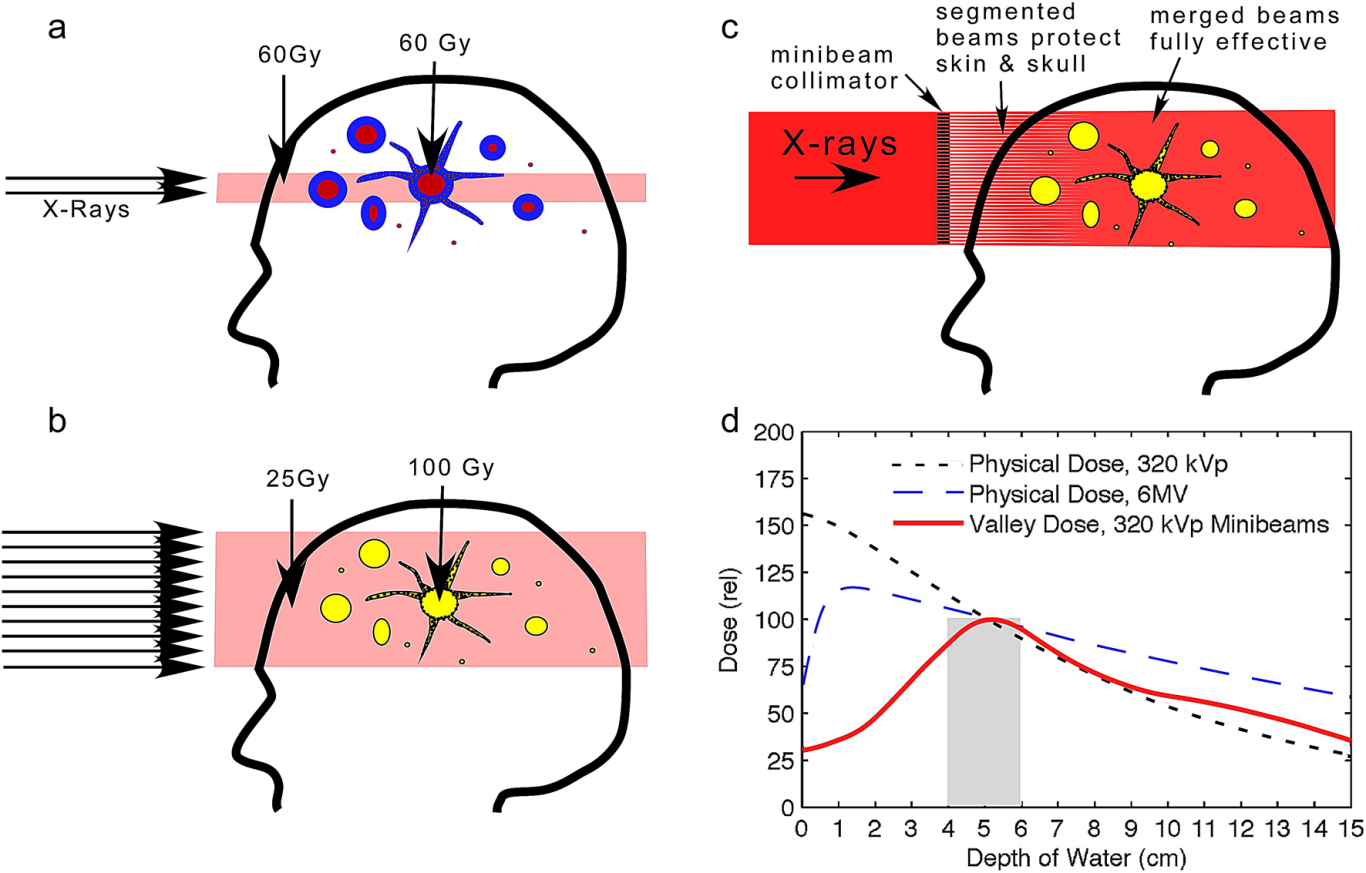
Current RT (**a**) aims ~ 60 Gy at a tumor growth but leaves many parts of the tumor (glioma with tentacle-like growth) or mets (with multiple lesions) untreated, leading to recurrence. We propose to lower the dose to a safer level for a larger volume irradiation, but boost the dose to effective levels only at tumor locations (**b**). (**c**): Use of a collimator to segment the beam allows tissue to recover from intervening unirradiated segments, which can then eliminate entrance dose damage. Once past the skin and skull the beams can be merged for effective therapy. (**d**): Monte Carlo simulations comparing unidirectional depth-dose distributions of 6 MV and 320 kVp X-rays with 320 kVp convergent minibeams. Figures (**a**) and (**b**) are from reference^7^ and (**d**) is from reference^33^. Although the 320 kVp energy spectrum is different from the 100 kVp used here (more appropriate for mice), this figure is only intended to present a concept adjusted to human use.

This effect suggests a new approach to tumors metastasizing to the brain. Instead of the current WBRT or stereotactic irradiation which are only temporarily palliative, WBRT could be combined with INPs to not only reduce the dose to normal brain, but elevate the dose to potentially ablative levels at the tumor lesions (Fig. 6a,b).

Physical dose enhancement is directly related to the absorption of X-rays by the high-Z element compared to tissue, and this varies with X-ray energy. For example, at the favorable K-edge of iodine (33 keV) K electrons are ejected and iodine absorbs ~ 119 times more than an equal weight of tissue. However at 10 MeV the absorption of X-rays by iodine drops to ~ 1/900th of what it is at 33 keV and is only ~ 1.8 times more than tissue absorption at that energy^34^. Therefore, the choice of X-ray energy and spectrum is critical to optimizing the dose enhancement^35^. A previous study found the optimal DEF for iodine versus water occurs at 50 keV^36^, which is higher than the K-edge since right at the K-edge the electron is ejected with no remaining kinetic energy for travel. With a standard tungsten target, the X-ray spectrum of a 100 kVp source (used in this study) has about 86% of its X-ray output intensity in the 33–80 kV range where iodine absorbs strongly and actually about 1.7 times better than gold^34,35,37^. The exact physical DEF by Monte Carlo calculation for the setup used here is beyond the scope of this paper, but other such studies found that 0.5% gold by weight produced a DEF of 1.8 using a 140 kVp X-ray source^26^. Perhaps a more relevant Monte Carlo study found that dose delivered to a human brain from a standard CT operating at 140 kVp produces a DEF of 2.35 for 0.5% iodine, higher than that found for gold. This can be explained by the higher absorption of iodine than gold for much of the X-ray spectrum from such sources. It should also be noted that the physical dose enhancement predicted can only be biologically realized if the ejected electrons and free radicals created can reach effective tumor targets. Further studies addressing this connection have shown that the heavy atom nanoparticle needs to be close to the nucleus^38^ or other sensitive targets such as mitochondria^39^. A confocal microlocalization study of the INPs indeed shows the INPs are close to many tumor nuclei^40^.

Specific targeting and localization of the INPs to brain tumors compared to normal brain is evident from the microCT images (Fig. 2). However, no active tumor targeting agent was used, such as an antibody or peptide. The localization may be explained by the small size of the INPs (20 nm), shown to have better tumor penetration than larger nanoparticles^41^, the Enhanced Permeability and Retention (EPR) effect^42^, nanoparticle transcytosis^43^, and the long blood half-life and tumor retention provided by the PEG coating^44^. Future incorporation of active targeting ligands may improve tumor loading or specificity.

The immune system is of paramount importance in cancer therapy. It is known that radiotherapy damages tumor cells exposing and creating new antigens that can heighten the immune response both locally and elsewhere and to prevent metastases via the abscopal effect. In the study here, athymic immunodeficient mice were used, which subtracted the role of active immunity in tumor control^45^. INP-RT might prove more effective in the context of an active immune system.

Chemotherapy is poorly effective when used to treat brain tumors. This is due to a number of factors including the tight blood brain and blood tumor barriers, dense extracellular matrix, and tumor efflux pumps^46^. By more effectively damaging the endothelial barrier and growing edge of tumors with INPs, chemotherapeutic drugs may be able to penetrate better and become more effective. We previously reported that INP-RT was synergistic with Doxil chemotherapy leading to some long-term survivors^7^. It is possible that drugs that were previously ineffective could be resurrected for an important benefit to patients with brain metastases when combined with INP-RT.

The RT used for this proof-of-principle study was provided as a single 15 Gy dose, reported to be approximately equivalent to 60 Gy given in 30 fractions^47^. However, RT is standardly fractionated. We have shown in a separate study that INP-RT is more effective than RT for fractionated RT comparing two doses of 10 Gy (one day apart) versus a single RT dose of 20 Gy. While the single 20 Gy RT dose provided greater life extension than two fractions, in both cases INP-RT was more effective than RT-only^40^. Also, the observation that the iodine in the INPs does not wash out from the tumor in at least three days^7^ appears promising for hypofractionation protocols now being used where the dose is delivered in one week^48^ since the INPs would not require multiple dosing.

In Fig. 3 the control group of INP only (no RT) was not done since earlier work showed INPs alone had no effect on tumor progression or survival^7^. However, that was for the U87 glioma model and not the 231 breast tumor. Future work should validate that INPs alone do not alter tumor progression for metastatic tumors.

In Fig. 4, the three longest surviving mice had at the start tumors ranked in size #4, 6, and 10 (10 being the largest). The average photon count for these 3 was 7.92E+07, which was larger than that of the whole group (6.40E+07). So these tumors were actually slightly larger than average and no correlation of survival with starting tumor size seems apparent.

Greater iodine tumor uptake and DEF was found 3 days after IV INP injection compared to that at 24 h (Table 1). The irradiation was done at 24 h to be consistent and for comparison with our previous experiments^7^. However, it is predicted that the results would be better if irradiated at 3 days, and preliminary data supports this^40^. Times less than 24 h were not studied here since uptake in gliomas showed lower tumor uptake at earlier times^40^.

The dose enhancement of iodine is largely dominated by the photoelectric effect which is mostly operative in the orthovoltage X-ray energy range (< 400 kV)^34–37^. In this range, tissue absorption is higher than with megavoltage X-rays. Absorption means better contrast imaging, so orthovoltage X-rays are used for CT, chest X-rays, interventional cardiology, orthopedic surgery, mammography, and also airport baggage inspection. Although at this energy X-rays do penetrate humans well, there is an exponential dose fall off with tissue depth. We used 100 kVp photons in this study, appropriate for mice due to their small size. However, for deep human tumors, radio oncologists have moved to megavoltage linacs (e.g., 6–25 MV) which avoid the high entrance dose (Fig. 6d). There are several viable solutions to retain the large iodine enhancement in the orthovoltage range in the clinical setting^7^: (a) convergent minibeams or microbeams, (b) CT irradiation, and (c) synchrotron RT. Synchrotrons operate in the orthovoltage range and are already being used to treat deep gliomas in patients after intravenous standard iodine contrast media, firing from several angles to reduce skin entrance dose^15,49^. A CT also has been used in a clinical trial^50^ after IV injection of rapidly clearing low molecular weight iodine contrast media. Monte Carlo modeling confirmed that standard CTs would be appropriate for human brain tumor therapy, especially with iodine loading^27^. Perhaps the most attractive method for practically using orthovoltage for deep tumors is provided by minibeam or microbeam technology. Minibeams use a multi-slit collimator to break the beam into evenly spaced segments of about 0.3 mm (Fig. 6c). Even smaller spatial beam segmentation (25–50 μm) with better tissue sparing is possible with the highly collimated X-rays produced by synchrotrons (Synchrotron Microbeam RT)^51–54^ since synchrotrons operate at high intensity enabling very short exposures which avoid merging of the segments that would otherwise occur due to tissue blood pulsations. For both mini- and microbeams it was found that blood vessels in the intervening unirradiated segments could repair the irradiated adjacent segments with no consequential residual damage, thus greatly reducing the entrance dose damage^55^. Also, by merging the segmented beams at the tumor, resulting in a damaging broad beam, deep tumors can be selectively irradiated, thus making even unidirectional orthovoltage irradiation superior to megavoltage^33,56^ (Fig. 6d). Minibeams or microbeams can also be used from multiple directions^57^ similar to gamma knife, or with intensity modulated RT (IMRT) for radiosurgery to focus the radiation on a specific tumor lesion. If INP-RT is shown to be far superior to standard RT of brain tumors in larger-animal trials such as dogs with spontaneous brain tumors, demand might grow for state of the art optimized kilovoltage irradiators to be built to take advantage of this potentially transformative technology.

## Conclusion

“Unfortunately, currently there is no effective clinical therapy for TNBCs”^58^. Sadly, this statement is applicable to all brain metastases. In this study we used intravenously delivered iodine nanoparticles with human TNBC tumors growing in the brains of athymic nude mice. High uptake in the tumor periphery was found by microCT, reaching 4.5% iodine by weight. This is predicted to yield a physical X-ray dose-enhancement factor of approximately 8, the highest ever achieved for any radiation dose-enhancing agent. In a radiotherapy trial, some mice with INP + RT experienced extraordinary life extensions with up to more than 10 times that provided by RT alone. Even better treatment of human brain metastases might result by combining INP-RT with synergistic chemotherapy and other therapies—such as immunotherapy and oncolytic virus therapy, using optimized irradiation equipment. INP-RT could provide a transformative new RT development to bring improved therapy to metastatic brain tumor patients where there are currently no fully effective alternatives.

## Methods

### Institutional animal assurance

Animal experiments were conducted according to NIH guidelines and approved by The University of Connecticut Health Center and Stony Brook University institutional animal care and use committees before start of the study.

### Tumor cells, tumor model and mice

MDA-MB-231 human triple negative breast cancer cells, obtained from the ATCC, were transduced with the pFULT vector, under the control of the human PGK1 promoter to express both luciferase and tomato, by the Skin Biology and Disease Resource-Based Center at Northwestern University, Chicago, Il. The mice were deeply anesthetized with intraperitoneal ketamine (140 mg/kg) and xylazine (3 mg/kg). After confirming deep anesthesia by toe pinch, a midline incision was made to expose the scalp. Using a hand-drill, a 0.5 mm wide burr hole was then drilled through the skull on the left coronal suture 2/3 of the way between the midline and the temporalis muscle insertion. Tumors were initiated by inoculating 1 μl of culture medium containing ~ one-hundred thousand luciferase and tomato expressing MDA-MB-231 cells, 2.5–3.0 mm deep into the left hemisphere of the mouse brain through the burr hole using a 27-gauge injector connected to a 1-μl Hamilton micro-syringe. Following a 1 min infusion of the cells, another 1 min was allowed for the cells to settle before removing the injector very slowly to minimally disturb the cells. The burr hole was then closed using surgical wax followed by skin closure using surgical glue^59^. U87 cells were implanted into the brain of athymic nude mice using the same method.

### Iodine nanoparticles

The synthesis and properties of the iodine nanoparticles were previously described^25^. Briefly, the INPs are 20 nm nanoparticles polymerized by crosslinking the standard clinically used triiodobenzene contrast agent iohexol and covalently adding a 1 K MW PEG coating. The blood half-life was found to be 40 h (2.7 days) and absence of toxicity at treatment doses determined by the absence of abnormal behavior, normal weight gain, the absence of abnormal clinical signs, normal complete blood work, and normal histopathology of major organs. Since the iodine is covalently bound, no abnormalities in thyroxine or thyroid enzyme levels were found^25^.

### IVIS

An In-Vivo Imaging System (IVIS Spectrum, Perkin Elmer) was used to quantify viable tumor cells. Imaging was done 13 min after a subcutaneous injection of luciferin (37.5 mg/kg). Photon counts were proportional to the number of luciferase transduced MDA-MB-231 tumor cells. A signal of ~ 8.0 × 10^8^ photon/sec corresponded to a tumor of ~ 2.5–3 mm diameter, which was confirmed by dissecting the brains of MDA-MB-231-luciferase-tomato tumor bearing mice. Parts of this procedure were previously described^7^.

### MicroCT imaging and quantification

Various times after IV injection (INPs at 70 mg iodine/ml), mice were imaged by microCT (Scanco Medical VivaCT 80, Bruttisellen, Switzerland), operated at 70 kVp. The source spot size was 5 µm (with 0.5 mm Al filtering), sampling with 15 × 15x15 µm voxels in a 30 mm-diameter field. 3 mm stacks of 200 sections at 2000 projections per revolution and an integration time of 300 ms/projection were collected, each stack requiring 20 min. Data were analyzed and viewed using Amira 3.1 software (Thermo Fisher Scientific, Waltham, Mass., USA).. Standards were prepared in tubes filled with a range of sodium iodide and Iohexol concentrations. Quantification was done by averaging the intensity over tissue volumes and reading the value from the standard curve adjusted for uninjected tissue values. Parts of these procedures were reported earlier^7,18,25^.

### Dose enhancement factor

Previous studies found the physical DEF^26,36^ was linear with concentration for gold or iodine and this relationship (DEF = 1.55 * conc by weight (%) + 1) was used to estimate the DEFs shown in Table 1.

### Treatment/irradiation

Tumors were grown to an advanced stage, reaching 5 × 10^8^–10^9^ photon counts assessed by IVIS. The group treated with INPs at 7.0 g I/kg were IV injected with a concentration of 70 mg I/ml INPs (4 injections, 2 each day spaced > 3 h apart). At 70 mg I/ml the INP solution was easily injected at a rate of about 0.5 mL over ~ 4 s using a 28 gauge needle without warming. Viscosity was acceptable at this concentration. Twenty-four hours after the last injection, mice were irradiated with 15 Gy X-rays. Mice were anesthetized with 140 mg/kg ketamine and 3 mg/kg xylazine in phosphate buffered saline given intraperitoneally in about 0.06 mL. The head and body were protected by a 3.4 mm-thick lead shield with a notch that enabled irradiation 8.0 mm caudally from the posterior canthus of the left eyelid and dorsally from the dome of the palate to above the calvarium. Irradiations used a Philips RT100 X-ray generator (Amsterdam, The Netherlands) operating at 100 kVp with a 1.7 mm Al filter. Dose was calibrated using a Radcal ion chamber (Monrovia, CA). To prevent lethal brain edema, dexamethasone (5 mg/kg) was injected subcutaneously 18 and again 6 h before irradiation and 6 and again 18 h after irradiation^60^.

## Data Availability

Explicit materials, data, and associated protocols are available from the corresponding author upon reasonable request.
